# Noise Pollution in Intensive Care Units and Emergency Wards

**Published:** 2011

**Authors:** Gholamreza Khademi, Masoumeh Roudi, Ahmad Shah Farhat, Masoud Shahabian

**Affiliations:** 1 *Department of pediatrics, Imam Reza Hospital, Mashhad University of Medical Sciences, Mashhad, Iran*; 2 *Department of audiology, Imam Reza Hospital, Mashhad University of Medical Sciences, Mashhad, Iran*; 3 *Department of pediatrics, Imam Reza Hospital, Mashhad University of Medical Sciences, Mashhad, Iran*; 4 *General physician, Mashhad University of Medical Sciences, Mashhad, Iran*

**Keywords:** Hospital, Intensive care units, Noise, Pollution, Sound

## Abstract

**Introduction:**

The improvement of technology has increased noise levels in hospital Wards to higher than international standard levels (35-45 dB). Higher noise levels than the maximum level result in patient’s instability and dissatisfaction. Moreover, it will have serious negative effects on the staff’s health and the quality of their services. The purpose of this survey is to analyze the level of noise in intensive care units and emergency wards of the Imam Reza Teaching Hospital, Mashhad.

**Procedure:**

This research was carried out in November 2009 during morning shifts between 7:30 to 12:00. Noise levels were measured 10 times at 30-minute intervals in the nursing stations of 10 wards of the emergency, the intensive care units, and the Nephrology and Kidney Transplant Departments of Imam Reza University Hospital, Mashhad. The noise level in the nursing stations was tested for both the maximum level (Lmax) and the equalizing level (Leq). The research was based on the comparison of equalizing levels (Leq) because maximum levels were unstable.

**Results:**

In our survey the average level (Leq) in all wards was much higher than the standard level. The maximum level (Lmax) in most wards was 85-86 dB and just in one measurement in the Internal ICU reached 94 dB. The average level of Leq in all wards was 60.2 dB. In emergency units, it was 62.2 dB, but it was not time related. The highest average level (Leq) was measured at 11:30 AM and the peak was measured in the Nephrology nursing station.

**Conclusion:**

The average levels of noise in intensive care units and also emergency wards were more than the standard levels and as it is known these wards have vital roles in treatment procedures, so more attention is needed in this area.

## Introduction

Noise is an accidental sound wave without any rhythm or harmony which can interfere with hearing. Noise pollution is defined as the level of environmental noise that is annoying. Noises higher than 85 dB cause hearing problems.

Even though noises with lower intensities do not lead to serious hearing problems, they can cause several harmful effects such as negative physiological consequences, deficient speaking awareness and recognition, and limited personal privacy. While the effects of noise in hospitals are not exactly known, there is no doubt that noise has different effects on patients’ and staffs’ well-being.

Continued presence of noise in the environment will cause an increase in the risk of high blood pressure and ischemic heart diseases in patients. It slows down healing and may contribute to patients’ aggressive behaviors as well (1-4).

Moor and his colleagues added that noise is the most important factor in provoking pain during the bedridden and post-surgical periods (3). Patients experiencing noise need more doses of sedatives than patients in a quiet environment.

Moreover, high levels of noise cause an increase in anxiety, masking vital alarms and reducing verbal communications, and may be an unseen added cause of mental stress for staff and on timely fatigue.

Based on the noise level, Pereira and his colleagues in a study classified the rate of noise in hospitals and according to this classification, the noise level in a quiet hospital is 40-50 dBA, in a hospital with moderate noise, it is 50-60 dBA and in a very noisy hospital, this noise level is 60-70 dBA (5-7).

It looks as if health procedures become much noisier with advances in technology. During the day, the average rate of noise increased from 57 dBs in 1960 to 72 dB in 2005 and the highest levels of noise in hospitals have reached 85 to 90 dB (8).

During the night, the noise level is worse than day and this high noise level continues through the weekend. In 1960, the average level of noise was 42 dB during the night, which went up to 60 dB in 2005.

According to the Global Health Organization’s recommendations, the maximum background noise should not be higher than 35 dB during the day and 30 dB at night (2).

Based on another survey, the highest level of noise should not be more than 40 dB, and higher levels lead to sleep disorders, cause stress and dissrupt communication skills (9).

The presence of noise pollution has destructive effects in departments which have emergency patients or procedures especially intensive care units. Because of the medical equipment, there is more noise pollution in such units. For example, telephones with ringtones, monitoring devices with sound alarms and infusion pumps cause noise by nature (1).

This is not only limited to adults’ ICU and emergency wards, but exists in newborn ICUs (where the lowest stresses bring about physiological changes such as apnea, decrease in oxygen levels, increased need for calorie and weight gain disorders. 

Due to the importance of noise pollution level in the ICU and emergency wards of the Imam-Reza Hospital and in order to recognize ways to reduce it, this study with the aim of determining the level of effective maximum noise levels in Imam the Reza Hospital has been performed.

This is a descriptive study which was done during the morning shifts in November-2009 at 30-minute intervals. 

In this study, the noise levels in the nursing stations of 10 wards in the Imam Reza Hospital were evaluated by using noise detector EXTECH 407727 (made by Extech Instruments, China), which measures sound levels between 35-110 dB. Prior to performing the study, to confirm the precision of the device, we calibrated the device based on standard instructions of the factory: range: 40-130 dB, microphone: one electret condenser type, accuracy 2±dB @94 dB/1000HZ). 

All measurements were read and recorded. Loud noises have negative effects on listeners even over short periods. Therefore, to study the instant noises in wards, we used the maximum sound level as well. 

To do so, we tested the Internal Disease Emergency, the Surgical Emergency, the Cardiovascular Emergency, CCU 1, CCU 2, the Internal ICU, the Neonatal ICU, the Open Heart ICU, the Nephrology and Kidney Transplant Depts. This study was done in a 30-day period and noise level measurements were performed for 2 minutes in each ward at 30-minute intervals for five days, overall 100 minutes for each ward. All evaluations were done by an experienced audiologist who was told to measure without alarming anyone to prevent changing their routine habits.

Results

The average equalized noise level in all wards was 60.24 dB and the highest point of the maximum noise level was 94 dB, which was seen in the Internal Disease (General) ICU at 8:30 in the morning. However, in other wards, it was not higher than 88 dB.

 In most wards, 9:30 to 11:00 during the morning was the peak of noise level. The Nephrology Unit and the Triple Emergency Units were found to be the noisiest places in different shift hours (Table 1).

The equalized noise levels in the Internal, Surgical and Cardiovascular Emergency Units were 62.9 dB, 62.4 dB and 62.2 dB, respectively. In this research, the noise level was higher than 60 dB in the Pediatric and Internal ICUs while it was only 59 dB in the Open Heart ICU, which was considered the quietest unit. The noisiest was the Internal ICU (61.3 dB). The time of peak level in ICUs was 9:30 a.m. In the NICU, the equalized level was 60.8 dB.

In CCU wards, this level was 54-56 dB, and the time of peak was 11:30 A.M (Fig 1).

**Table 1:** Ward-time Leq measurements

**AM** **)** **time)**	7.3.0	8	8.30	9	9.30	10	10.30	11	11.30	12
**ward**										
General Emergency	63	59	61	61	62	63	65	66	60	60
Surgical Emergency	60	58	63	62	66	65	66	63	58	63
Cardiovascular Emergency	54	57	67	66	63	63	66	58	65	63
CCU1	60	57	55	56	55	56	54	60	61	55
CCU2	54	55	52	53	53	52	56	57	62	56
General ICU	55	59	62	62	60	59	63	58	62	64
NICU	54	58	62	89	66	58	65	64	62	60
ICU	57	60	60	58	59	62	61	60	58	59
Nephrology	58	66	60	65	71	67	56	63	65	66
Transplant unit	55	48	57	54	56	59	59	62	56	60

**Diagram 1:**
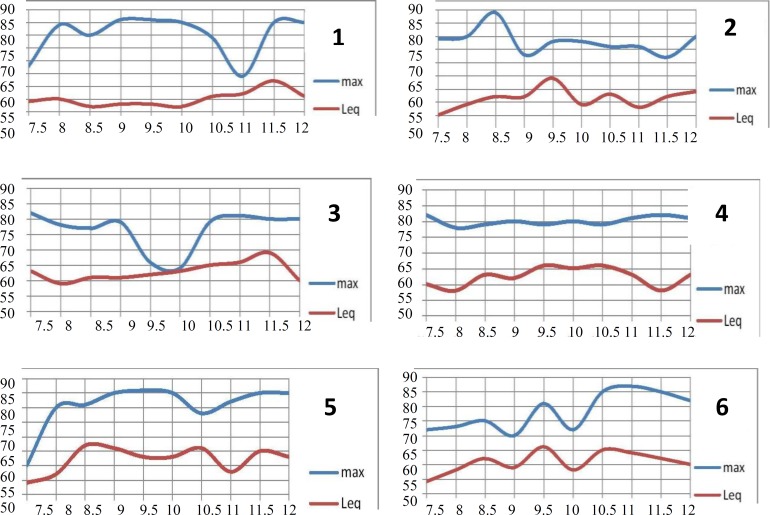
Lmax and Leq at different times in Intensive Care Units & Emergency Wards: (1) CCU 2, (2) General ICU, (3) Internal Emergency, (4) Surgical Emergency, (5) Cardiovascular Emergency, (6) NICU, (7) Open Heart Surgery ICU, (8) CCU 1, (9) Nephology, and (10) Kidney Transplant Unit

**Diagram 2:**
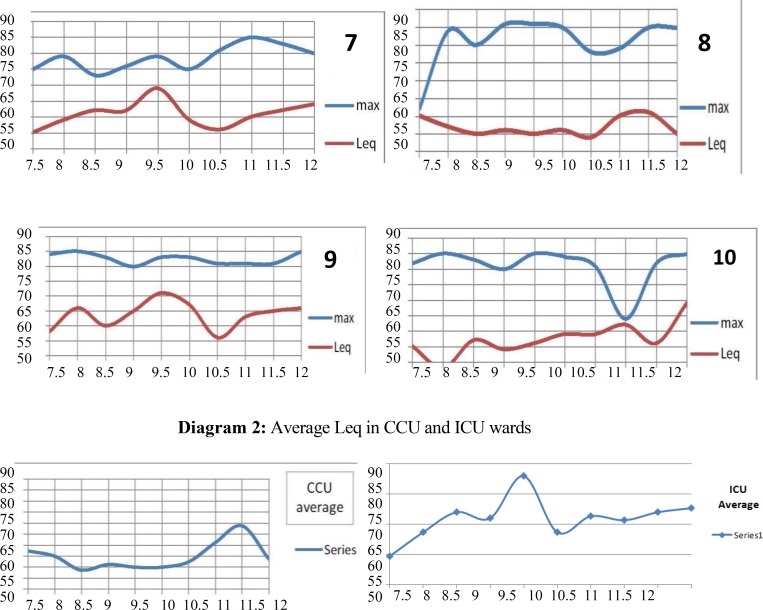
Average Leq in CCU and ICU wards

## Discussion

Technology development has increased noise levels in hospital environments above the international standards (about 35-45 dB) (1,2). 

Increased noise levels beyond standard limits will lead to serious effects on patients’ treatment and also on the quality of personnel’s services.

In this research the Nephrology Department had the highest level of noise, followed by the Emergency Department. The average level of evaluated noise in all units was 60.24 dB, which is higher than the standard average of the World Health Organization. Although noise levels lower than 85 dB do not lead to hospital staff deafness (10), such levels show a non-standard environment, which is much more important in ICU units.

The average noise levels in internal, surgical and cardiovascular emergency units were 62.9 dB, 62.4 dB and 62.2 dB, respectively. According to the emergency procedures of these units, there was no particular time index. In ICUs, noises may be produced by ventilators, infusion pumps and cardiac monitors, which are vital in improving the patient’s condition. Telephone ringtones, and medical beeping instruments and alarms are tiring and bring about irritation to patients and probably lead to doctors’ mistakes and may even increase the death rate (11).

Some studies have shown that normal sleeping problems of the staff is due to ICU noises (4). 

In a research in Greece, the noise level in ICUs was reported to be 27 dB more than standard limits (67 dB) (12). 

In another research in ICU units, the time of noise pollution of each instrument was separately examined, which showed that the main cause of noise pollution of ICU environments was television (23%). Cardiac monitor alarms were in the second place. Based on this research, which was done by Douglas, et al, television and people speaking in the environment account for 49 percent of noise (13).

Although the Open Heart Surgery Ward was considered the quietest place, the noise level was 59 dBA, which is also far beyond the standard levels. 

The noisiest ICU was the Internal ICU. The maximum of Leq in this ICU is around 9:30 A.M.

In the NICU, the average noise level was 60.8 dB, which was mainly caused by from personnel’s talking and also patients’ relatives speaking with each other. We saw an increase when babies cried and also when heart monitors alarms were set off. The noise level in the feeding room of babies should not exceed 35-55 dBA. 

In our study, there was a significant difference between the current situations in the NICU and the standard conditions, and this pollution causes stress in babies, which results in physiological changes such as apnea, bradycardia, a decrease in the temporary pressure of oxygen and an increase in the required calories, which results in difficulty in babies gaining weight and also results in the lowering of neurological skills of the baby. 

In our CCU study, the average sound level in all wards during the day was almost the same, except at 11:30 due to patients’ relatives speaking. A sudden sound in our study is 90 dBA. 

In a research, the noise level in the CCU was recorded and was propagated for a preliminary group during their sleep, it caused sleep disorders. These disorders were as follow: shortened sleep time, increased wakening time, difficulty in falling sleep and poor quality of sleep (14). 

If we compare our study with the survey of Pereira et al, which classifies hospitals according to their levels of noise, we can classify the Imam Raze Hospital as a noisy hospital. 

But what can be done to reduce these noise levels? The most important thing is to control the noise level of the instruments before installation. The devices used in hospitals should have low noise levels. 

Noise control in hospitals is very important. 

We should, therefore, encourage patients and personnel to use simple methods such as closed doors or lower tones to decrease the noise level. In a study performed by Monsen et al in a neurology ICU, an educational conference on the topic of Methods of Reducing Noise Pollution was performed for personnel. This eventually led to a dramatic reduction in noise level in the ICU (15). 

On the Contrary, Pilibin and Gary claimed that change in Staffs behavior has no significant effect on the control of sound pollution in hospital words. However, more important effect is associated to physical changes that must be done in these wards (16). 

According, in another study performed in an oncology department, researchers succeeded in reducing the noise level up to 50 dB by installing acoustic fiberglass sheets on walls and ceilings (17). 

In addition, in another study some interventions were performed to reduce the noise level of nightshifts in surgical ICU such as: reducing the sound of alarms, low-voiced conversation, no use of telephone ring, radio and television (18).

## Conclusion

The sound level in all Imam Reza Hospital Wards evaluated in is study, was beyond the standard limit. Nephrology department and triple emergency ward were considered the noisiest places in different hours of the shift. The maximum point of the sound level was between q: 30 to N: 30 This research must be one the other department and in other shift times and the staff of treatment team must be taught how to reduce the controllable noises. Equipments and standard procedures must be used to reduce the noise level in different hospital wards as well.

## References

[1] Rabiyan M, Gharib M (2003). Teb va Tazkiye.

[2] World Health Organization Occupational and community noise.

[3] Moore MM, Nguyen D, Nolan SP (1998). Interventions to reduce decibel levels in patient care units. Am Surg.

[4] Otenio MH, Cremer E (2007). Noise level in a 222 bed hospital in the 18th health region-PR. Rev Bras Otorrinolaringol.

[5] Pereira RP, Toledo RN, Amaral JLG, Guilherme A (2003). Rev Bras Otorrinolaringol.

[6] Busch-Vishniac IJ, West JE, Barnhill C, Hunter T, Orellana D, Chivukula R (2005). Noise levels in Johns Hopkins Hospital. J Acoust Soc Am.

[7] Stokowski LA The inhospitable hospital: No peace, No quiet.

[8] MacKenzie DJ, Galbrun L (2007). Noise levels and noise sources in acute care hospital wards. Build Serv Eng Res Technol.

[9] Correa LC, Zago SBA, Posso SBM, Criollo TC (2004). Rev Univap.

[10] Macedo IS, Mateus DC, Asprino AC, Lourenço EA (2009). Noise assessment in intensive care units. Braz J Otorhinolaryngol.

[11] Giusti GD, Piergentili F (2009). Noise in the intensive care unit: A summary review. World Crit Care Nurs.

[12] Tsiou C, Eftymiatos D, Theodossopoulou E, Notis P, Kiriakou K (1998). Noise sources and levels in the Evgenidion Hospital intensive care unit. Intensive Care Med.

[13] Kahn DM, CookTE, Carlisle CC, Nelson DL, Kramer NR, MillmanRP (1998). Identification and modification of environmental noise in an ICU setting. Chest.

[14] Topf M, Bookman M, Arand D (1996). Effects of critical care unit noise on the subjective quality of sleep. J Adv Nurs.

[15] Monsen MG, Edell-Gustafsson UM (2005). Noise and sleep disturbance factors before and after implementation of a behavioral modification program. Intensive Crit Care Nurs.

[16] Philbin MD, Gray L (2002). Changing levels of quiet in an intensive care nursery. J Perinatol.

[17] MacLeod M, Dunn J, Busch-Vishniac IJ, West JE, Reedy A (2007). Quieting Weinberg 5C: A case study in hospital noise control. J Acoust Soc Am.

[18] Walder B, Francioli D, Meyer JJ, Lancon M, Romand JA (2000). Effects of guidelines implementation in a surgical intensive care unit to control night time light and noise levels. Crit Care Med.

